# Transcriptomic Analysis of High- and Low-Virulence Bovine *Pasteurella multocida in vitro* and *in vivo*

**DOI:** 10.3389/fvets.2021.616774

**Published:** 2021-02-10

**Authors:** Fang He, Zongling Zhao, Xiaoyan Wu, Lijie Duan, Nengzhang Li, Rendong Fang, Pan Li, Yuanyi Peng

**Affiliations:** ^1^College of Veterinary Medicine, Southwest University, Chongqing, China; ^2^Guangdong Laboratory of Lingnan Modern Agriculture, Guangdong Provincial Key Laboratory of Animal Nutrition Control, National Engineering Research Center for Breeding Swine Industry, College of Animal Science, South China Agricultural University, Guangzhou, China

**Keywords:** *Pasteurella multocida*, transcriptomic analysis, virulence genes, *in vitro*, *in vivo*

## Abstract

*Pasteurella multocida* is a gram-negative opportunistic pathogen that causes various diseases in poultry, livestock, and humans, resulting in huge economic losses. *Pasteurella multocida* serotype A CQ6 (PmCQ6) is a naturally occurring attenuated strain, while *P. multocida* serotype A strain CQ2 (PmCQ2) is a highly virulent strain isolated from calves. Compared with PmCQ2, it was found that bacterial loads and tissue lesions of lung tissue significantly decreased and survival rates significantly improved in mice infected with PmCQ6 by intranasal infection. However, comparative genome analysis showed that the similarity between the two strains is more than 99%. To further explore the virulence difference mechanism of PmCQ2 and PmCQ6, transcriptome sequencing analysis of the two strains was performed. The RNA sequencing analysis of PmCQ2 and PmCQ6 showed a large number of virulence-related differentially expressed genes (DEGs) *in vivo* and *in vitro*. Among them, 38 virulence-related DGEs were significantly up-regulated due to PmCQ6 infection, while the number of PmCQ2 infection was 46, much more than PmCQ6. In addition, 18 virulence-related DEGs (capsule, iron utilization, lipopolysaccharide, and outer membrane protein-related genes) were up-regulated in PmCQ2 infection compared to PmCQ6 infection, exhibiting a higher intensive expression level *in vivo*. Our findings indicate that these virulence-related DEGs (especially capsule) might be responsible for the virulence of PmCQ2 and PmCQ6, providing prospective candidates for further studies on pathogenesis.

## Introduction

*Pasteurella multocida* is a gram-negative bacterium well-known for causing diseases in a wide range of birds and mammals (e.g., humans and economically important animal species) (1), including fowl cholera, swine atrophic rhinitis, rabbit septicemia, bovine pneumonia, and human wound abscesses/meningitis following cat/dog-inflicted injuries (2–5), which lead to huge economic losses to animal husbandry worldwide. *Pasteurella multocida* have been classified into five serotypes (A, B, D, E, and F) according to the specificity of capsular antigens (6, 7) and 16 Heddleston serotypes based on the lipopolysaccharide (LPS) antigens (8). The capsular type A *P. multocida* mainly causes pneumonia and serious bovine respiratory diseases.

*Pasteurella multocida* possesses a number of virulence factors, mainly including capsular polysaccharide, iron utilization-related genes, LPS, and outer membrane proteins (OMPs) (4, 9, 10). It is well-known that the virulence factors expressed by *P. multocida* play key roles in pathogenesis (11–13). For example, the virulence of *P. multocida* acapsular mutants was strongly attenuated in mice (14); the *galE* (UDP-galactose) mutant for *P. multocida* LPS biosynthesis was completely attenuated in mice (15).

PmCQ2 and PmCQ6 were previously isolated from the pneumonic lungs of two calves (16, 17). PmCQ2 shows high lethality, while PmCQ6 exhibits low toxicity. Recently, we have reported that NLRP3 inflammasome plays an important role in caspase-1 activation and IL-1β secretion in macrophages infected with *P. multocida* (18); PmCQ2 and PmCQ6 induced differential NLRP3 inflammasome activation and subsequent IL-1β secretion (17). Moreover, our previous study found that there is 99% genome similarity with high collinearity between PmCQ2 (2,002 protein-coding genes) and PmCQ6 (1,970 protein-coding genes) (19). Additionally, the bacterial loads and six known/potential virulence genes (*ompA, ompH, pfhB2, hasR, pm0979*, and *pm0442*) of PmCQ2 in mice were significantly higher than that of PmCQ6 (20). However, the transcription of other virulence genes is still unclear.

In this study, we demonstrated that, compared with PmCQ2, the bacterial loads and tissue lesions of lungs significantly decreased, and the survival rates significantly improved in mice infected by PmCQ6. Notably, the transcriptome sequencing analysis of PmCQ2 and PmCQ6 showed a large number of DEGs *in vivo* and *in vitro*. A functional analysis of these DEGs demonstrated that capsule-, iron utilization-, LPS-, and OMP-related virulence genes were enriched. The results indicate that these virulence-related DEGs might be responsible for the virulence differences of PmCQ2 and PmCQ6, providing a new insight of infection mechanism to *P. multocida*.

## Materials and Methods

### Bacterial Strains and Culture Conditions

The low-virulence bovine *P. multocida* serotype A strain CQ6 (PmCQ6) and highly virulent bovine *P. multocida* serotype A strain CQ2 (PmCQ2) were isolated from the lungs of calves with pneumonia in Chongqing, China (21). The strains were streaked on Martin agar plates (Qingdao Hope Biol-Technology Co., Ltd., Qingdao, China) and incubated at 37°C for 24 h, and one colony of each strain was inoculated into 5 mL Martin broth and cultured for 12 h at 37°C with shaking (220 revolution/min).

### Experimental Animals and Ethics Statement

A total of 130 C57BL/6 female mice (7–8 weeks old) were purchased from Hunan SJA Laboratory Animal Co., Ltd. (Hunan, China). The mice were housed in individually ventilated pathogen-free cages (temperature at 20–30°C, relative humidity at 50–60%, and lighting cycle at 12 h/day) with free access to food and water. The mice were acclimated for 4 days after arrival before starting the experiments. All animal experiments were carried out with approval from the Animal Ethics and Research Committee of Southwest University (Permit No. 11-1025), Chongqing, China.

### Pathogenicity of PmCQ2 and PmCQ6

To determine the pathogenicity of PmCQ2 and PmCQ6, C57BL/6 mice were infected by intranasal exposure with PmCQ2 and PmCQ6 at a dose of 1 × 10^8^ colony-forming units (CFU) in 20 μL, and the number of mice used for each strain detection was equal. The mice were monitored for 7 days to determine the survival curves, and mice showing severe clinical signs (e.g., depression, accelerated breath, cough, hairiness, and lethargy) were considered moribund and were humanely killed. Survival rates (*n* = 10/group) were measured in the two groups after injection. The mice were also euthanized for collection of lung tissues to measure the bacterial loads (*n* = 10/group) and histopathological examination (HE; *n* = 6/group) at 16 h post-infection.

### Pathological Examinations

For histopathological examination, the HE experiments were performed as described in the previous study (22). The lung tissues (*n* = 6/group) were immediately fixed in 4% paraformaldehyde for 24 h, dehydrated in graded ethanol, and then embedded in paraffin wax. The tissues were sliced at 3 μm thickness and then stained with hematoxylin and eosin (H&E) (16). According to our previous method, briefly, histopathological scoring was performed by a pathologist blinded to the PmCQ2 and PmCQ6 groups, and the scoring was mainly based on interstitial inflammation, vascular endothelialitis, bronchitis, edema, serous effusion, and thrombus formation. All parameters were scored separately from 0 (lesion absent) to 3 (severe lesion) (23).

### Bacterial Colonization

To measure the bacterial loads, lung tissues (*n* = 10 in each group) were collected at 16 h post-bacterial infection. The tissues were homogenized aseptically, and bacterial loads were quantified by 10-fold serial dilution in saline. These different dilutions were plated in triplicate on Martin's broth agar and were incubated at 37°C for 24 h to count the CFU.

### The Sample Preparation of Transcriptome Analysis

C57BL/6 mice were infected by intranasal inoculation with PmCQ2 (*n* = 3) and PmCQ6 (*n* = 3) at a dose of 1 × 10^8^ CFU in 20 μL. The mice were euthanized at 16 h post-infection; lung tissues were collected and quickly frozen in liquid nitrogen. Simultaneously, 1 mL each of fresh mid-logarithmic phase of PmCQ2 (*n* = 3) and PmCQ6 (*n* = 3) was seeded in 100 mL fresh Martin liquid medium and incubated at 37°C with shaking at 220 revolution/min for 12 h. The bacterial cultures were centrifuged at 3,000 revolution/min for 10 min at 4°C; the bacterial cell pellets were suspended in 10 mL ice-cold phosphate-buffered saline (PBS) and centrifuged again. The bacterial cells were quickly frozen in liquid nitrogen after washing three times. The lung tissues and bacteria samples were sent to Beijing Genomics Institute (BGI, Shenzhen, China) for transcriptome sequencing.

### RNA Isolation, Library Construction, and Illumina Deep Sequencing

Total RNA was extracted using Trizol reagent (Invitrogen Life Technologies, USA), following the manufacturer's protocol. RNA integrity was confirmed by agarose gel electrophoresis and quantified by NanoDrop (NanoDrop 2000, Thermo Scientific). Subsequently, biotin-labeled specific probes were used to remove rRNA, and then RNA is purified and fragmented. Then, 1 μg of each RNA sample was used to construct a strand-specific cDNA library according to the recommendations of Illumina TruSeq Stranded Kit. The cDNA library size was analyzed by using an Agilent 2100 Bioanalyzer, and the effective concentration was determined using qPCR (StepOnePlus Real-Time PCR Systems, Thermo Scientific). Finally, the samples were sequenced on Illumina sequencing platform (HiSeq 4000), and 150-bp paired-end reads were generated.

### Reads Filtering and Mapping, Qualification, and Identification of Differently Expressed Genes

The reads are aligned to the rRNA reference sequence *via* SOAP (24), and these reads are removed. Reads with an average base quality lower than Q20, unknown base N content >5%, and containing linkers (linker contamination) were discarded. The reads were mapped to the genome by *HISAT* software (25). The clean reads were mapped to the reference gene set by using *Bowtie2* software (26) (http://bowtie-bio.sourceforge.net/Bowtie2/index.shtml), and then *RSEM* software (27) (http://deweylab.biostat.wisc.edu/RSEM) was used to calculate the gene expression levels. Differential expression analysis was performed using estimateSizeFactors in DESeq (version 1.18.0) R package; *p*-value and fold change value were identified using nbinomTest in DESeq with R package. *P* < 0.05 and |log2foldchange| ≥ 1 were set as the threshold for DEG identification. The hierarchical cluster analysis of DEGs was performed by clustering software *cluster* (28, 29). The data in this study have been deposited to NCBI's Sequence Read Archive database, and the accession numbers are PRJNA597831, PRJNA629108, and PRJNA664837.

### Quantitative Real-Time PCR

All RNA extractions were performed using an RNAprep pure animal/cell/bacteria kit (Tiangen, Beijing, China) involving a gDNA elimination step. cDNAs were synthesized with an iScript cDNA synthesis kit (Bio-Rad, California, USA), and quantitative real-time RT-PCR was performed according to previous study using a CFX96 instrument (Bio-Rad, California, USA) (16). Each target gene was individually normalized to the reference gene 16S by using the quantification method 2^−ΔΔCt^ (30). Specific primers were designed according to the reference sequences in NCBI with Primer-BLAST under the criteria: (a) PCR product size 70–200 bp and (b) melting temperature 60 ± 2°C. The qRT-PCR primer sequences are listed in Supplementary Table 1.

### Quantification of Hyaluronic Acid in the Capsule of Bacteria

According to the method described previously (23), bacteria were grown in 100 ml fresh Martin liquid medium and incubated at 37°C with 220 revolution/min for 8 h. Then, the culture was centrifuged at 7,600 g for 15 min, and the supernatant was removed. Next, the bacterial cells were washed twice with PBS. The bacteria were then re-suspended in PBS and incubated for 1 h at 42°C. At the same time, the number of bacteria was counted on Martin agar plates before and after incubation at 42°C. The bacterial solutions were centrifuged, and the supernatant was collected for the detection of capsule content based on our previous description (31). Briefly, 10-μL sample or 10 μL hyaluronic acid standards were added to 90 μL capsule staining solution (0.2 g/mL stain in all staining solutions, 0.06% glacial acetic acid in 50% formamide). After mixing, OD640 was determined by a microplate reader, and the capsule content was calculated.

### Statistical Analysis

All data were displayed as means ± standard deviation (SD). GraphPad Prism 6.0 software was applied for all statistical analysis. The survival rates of mice were evaluated using Kaplan–Meier analysis. Data between the two groups were analyzed using unpaired *t*-tests if the data were in Gaussian distribution and had equal variance or by unpaired *t*-test with Welch's correction if the data are in Gaussian distribution but show unequal variance or by non-parametric test (Mann–Whitney *U*-test) if the data were not normally distributed. The Gaussian distribution of data was analyzed by D'Agootino–Pearson omnibus normality test and Kolmogorov–Smirnov test. The variance of data was analyzed by Brown–Forsythe test. Significant differences were considered at *P* < 0.05 (^*^P < 0.05, ^**^P < 0.01, ^***^P < 0.001).

## Results

### Comparison of Pathogenicity Between PmCQ2 and PmCQ6

**Figure 1:**
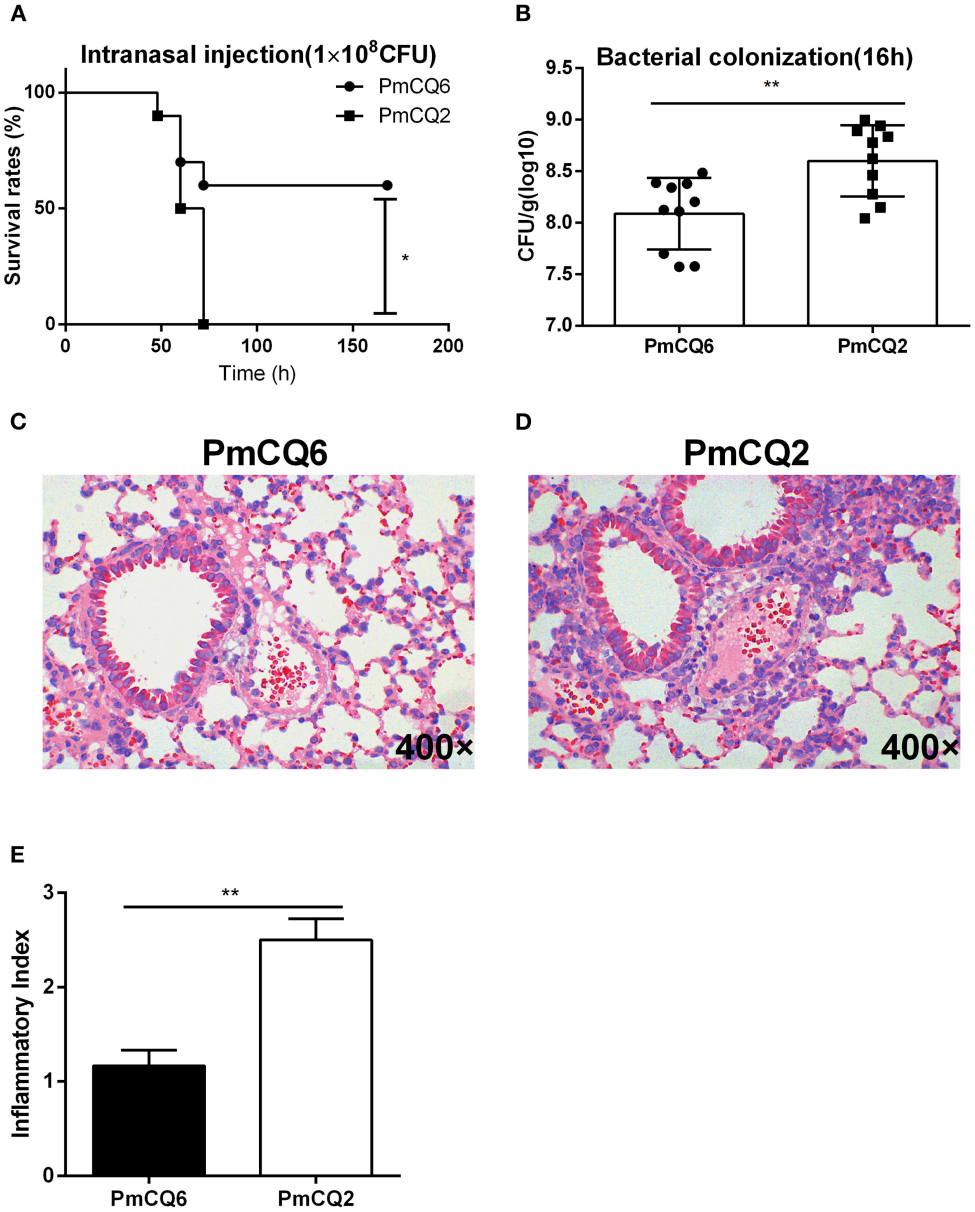
Mice model infected with PmCQ2 and PmCQ6. **(A)** Survival curve of mice challenged with PmCQ2 and PmCQ6 by intranasal injection (2 × 10^8^ CFU). The data were representative of three independent experiments with 10 mice per group and were analyzed by Kaplan–Meier analysis (*p < 0.05). **(B)** Bacterial burden comparison of mice lungs infected with PmCQ2 and PmCQ6 at 16 h. The data were representative of three independent experiments with 10 mice per group. The data were analyzed with unpaired *t*-test and expressed as means ± SD (**p < 0.01). **(C–E)** The pulmonary histopathological examination of mice at 16 h, stained with H&E (magnification ×400). The data were representative of two independent experiments with six mice per group. The data were analyzed with Mann–Whitney *U*-tests and expressed as means ± SD (**p < 0.01).

In mice infected with PmCQ6 (1 × 10^8^ CFU) by intranasal injection and observed for 1 week, 60% of them survived, while the PmCQ2 (1 × 10^8^ CFU)-infected mice all died rapidly within 72 h (Figure 1A). The bacterial burden of PmCQ2 (1 × 10^8^-1 × 10^9^ CFU) in mice lungs was significantly higher than that of PmCQ6 (1 × 10^7.5^-1 × 10^8.5^ CFU) at 16 h (Figure 1B). Compared with the PmCQ6-infected group, the lung tissue lesions (especially inflammatory cell infiltrates) in the PmCQ2-infected group also showed a significant increase based on H&E staining (Figures 1C–E).

### DEGs Identification of PmCQ2 and PmCQ6 *in vitro* and *in vivo*

**Figure 2:**
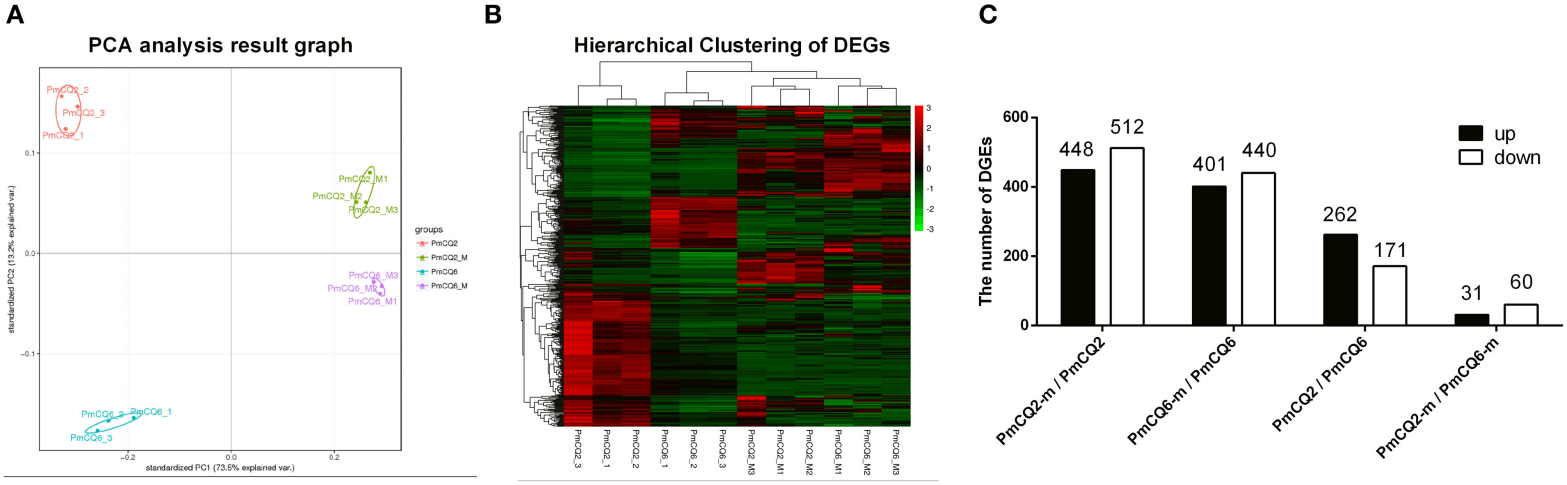
The results of RNA sequencing analysis. **(A)** Principal component analysis of PmCQ2 and PmCQ6 *in vitro* and *in vivo*. **(B)** Heat map for clustering of differentially expressed genes (DEGs) of PmCQ2 and PmCQ6 *in vitro* and *in vivo*. **(C)** The up/down-regulated DEGs of PmCQ2-m/PmCQ2, PmCQ6-m/PmCQ6, PmCQ2/PmCQ6, and PmCQ2-m/PmCQ6-m.

All data about the quality of transcriptomic sequencing are shown in Supplementary Table 2. The average percentages mapped to the reference genome were 92.28, 90.32, 1.96, and 0.57% in PmCQ2, PmCQ6, PmCQ2-m, and PmCQ6-m, respectively. Based on the transcriptome analysis of PmCQ2 and PmCQ6 *in vitro* and *in vivo* (fold change ≥ 2, *P*-value ≤ 0.05), a total of 960 DEGs were observed in PmCQ2-m (*in vivo*)/PmCQ2 (*in vitro*), including 448 up-regulated and 512 down-regulated genes; there were 841 DEGs (401 up-regulated genes and 440 down-regulated genes) in PmCQ6-m (*in vivo*)/PmCQ6 (*in vitro*); 433 DEGs, with 262 up-regulated genes and 171 down-regulated genes, were in PmQ2/PmCQ6; and 91 DEGs were in PmQ2-m/PmCQ6-m, of which 31 were up-regulated and 60 were down-regulated (Figures 2A–C and Supplementary Table 3). Each sample was normalized with fragments per kilobase of transcript per million reads mapped or reads per kilobase of transcript per million reads mapped, and the expression level was displayed in Supplementary Table 4.

### Virulence-Related DEGs of PmCQ2 and PmCQ6 *in vitro*

**Figure 3:**
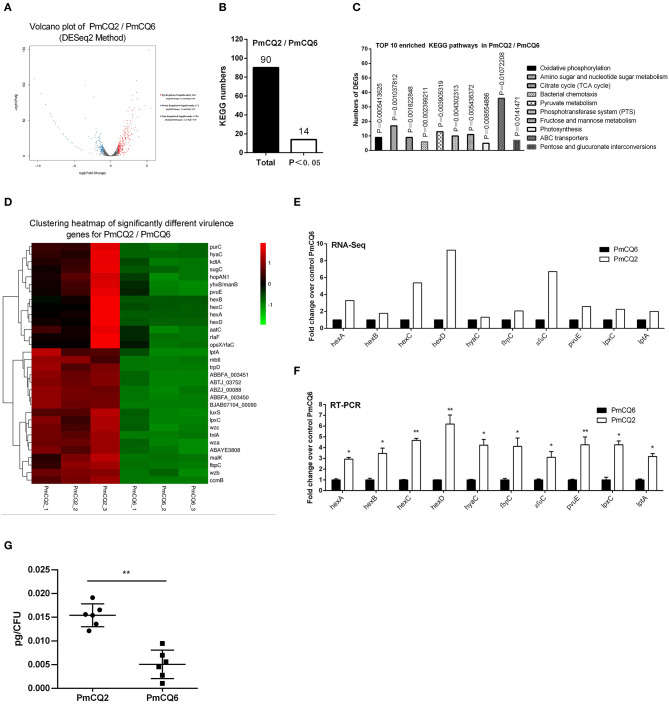
Virulence factor-related differentially expressed genes (DEGs) of PmCQ2 compared to PmCQ6. **(A)** Volcano plot of PmCQ2/PmCQ6. **(B)** Total Kyoto Encyclopedia of Genes and Genomes (KEGG) numbers and significant KEGG numbers in PmCQ2/PmCQ6. **(C)** Top 10 enriched KEGG pathways of PmCQ2/PmCQ6, and the number of DEGs in each pathway is counted. **(D)** The clustering heat map of virulence factor-related DEGs in PmCQ2/PmCQ6. **(E)** The mRNA expression (RNA-Seq) of virulence factor-related DEGs in PmCQ2/PmCQ6 (*n* = 3). **(F)** The mRNA expression (RT-PCR) of virulence factor-related DEGs in PmCQ2/PmCQ6 (*n* = 3). **(G)** The content of capsule polysaccharide in PmCQ2 and PmCQ6. **(F)** is representative of two independent experiments with three replicates per group and analyzed by multiple comparative analyses. **(G)** is representative of two independent experiments with six replicates per group and was determined by unpaired *t*-test. All data were expressed as means ± SD (*p < 0.05, **p < 0.01).

The RNA sequence analysis of the virulent PmCQ2 and attenuated PmCQ6 *in vitro* (Figure 3A) showed that 433 DEGs were identified. By KEGG enrichment analysis, a total of 90 KEGG pathways were matched, and 14 KEGG pathways were significantly enriched (*P* < 0.05; Figure 3B and Supplementary Table 5). The top 10 enriched KEGG pathways are shown in Figure 3C, including oxidative phosphorylation, amino sugar and nucleotide sugar metabolism, citrate cycle (TCA cycle), bacterial chemotaxis, pyruvate metabolism, phosphotransferase system (PTS), fructose and mannose metabolism, photosynthesis, ABC transporters, and pentose and glucuronate interconversions. Moreover, 32 virulence factors related to fifteen capsular polysaccharides, six iron utilization, four LPS, and seven others were significantly up-regulated in PmCQ2/PmCQ6 (Figure 3D and Supplementary Table 6). To validate the results of the RNA sequence analysis, five capsular polysaccharide-related genes (*hexA/B/C/D, hyaC*), three iron utilization-related genes (*fbpC, afuC, pvuE*), and two LPS-related genes (*lpxC, lptA*) were chosen for qPCR analysis, which were similar to those obtained from the RNA-seq results (Figures 3E,F). In addition, the capsular content of PmCQ6 was lower than that of PmCQ2 (Figure 3G).

### Virulence-Related DEGs of PmCQ2 and PmCQ6 *in vivo* and *in vitro*

**Figure 4:**
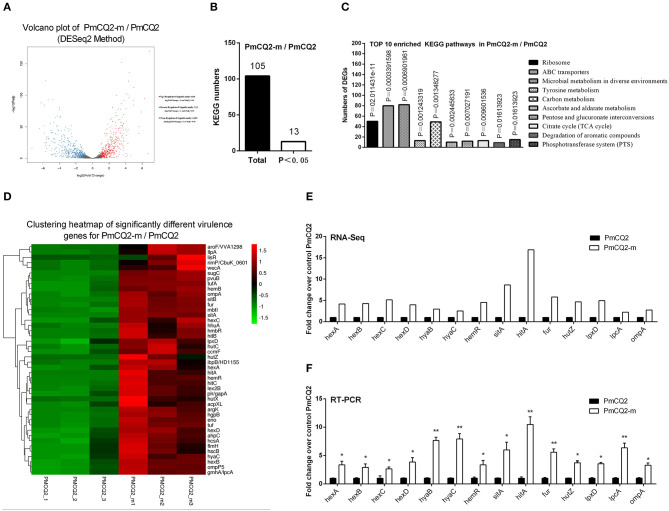
Virulence factor-related differentially expressed genes (DEGs) of PmCQ2-m compared to PmCQ2. **(A)** Volcano plot of PmCQ2-m/PmCQ2. **(B)** Total Kyoto Encyclopedia of Genes and Genomes (KEGG) numbers and significant KEGG numbers in PmCQ2-m/PmCQ2. **(C)** Top 10 enriched KEGG pathways of PmCQ2-m/PmCQ2, and the number of DEGs in each pathway is counted. **(D)** The clustering heat map of virulence factor-related DEGs in PmCQ2-m/PmCQ2. **(E)** The mRNA expression (RNA-Seq) of virulence factor-related DEGs in PmCQ2-m/PmCQ2 (*n* = 3). **(F)** The mRNA expression (RT-PCR) of virulence factor-related DEGs in PmCQ2-m/PmCQ2 (*n* = 3). These were representative of two independent experiments, with three replicates per group, analyzed by multiple comparative analysis and expressed as means ± SD (*p < 0.05, **p < 0.01).

**Figure 5:**
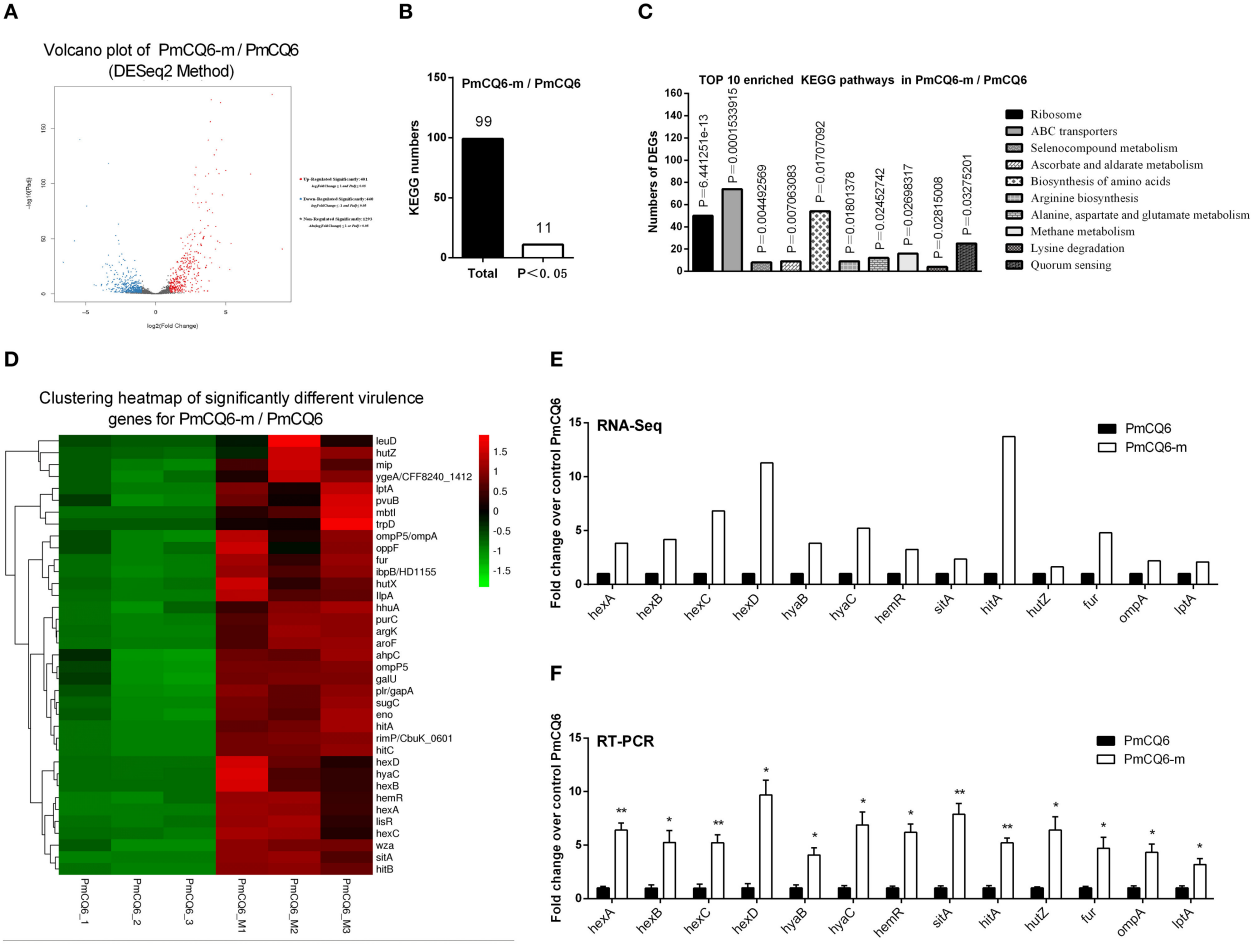
Virulence factors-related differentially expressed genes (DEGs) of PmCQ6-m compared to PmCQ6. **(A)** Volcano plot of PmCQ6-m/PmCQ6. **(B)** Total Kyoto Encyclopedia of Genes and Genomes (KEGG) numbers and significant KEGG numbers in PmCQ6-m/PmCQ6. **(C)** Top 10 enriched KEGG pathways of PmCQ6-m/PmCQ6, and the number of DEGs in each pathway is counted. **(D)** The clustering heat map of virulence factor-related DEGs in PmCQ6-m/PmCQ6. **(E)** The mRNA expression (RNA-Seq) of virulence factor-related DEGs in PmCQ6-m/PmCQ6 (*n* = 3). **(F)** The mRNA expression (RT-PCR) of virulence factor-related DEGs in PmCQ6-m/PmCQ6 (*n* = 3). These were representative of two independent experiments, with three replicates per group, analyzed by multiple comparative analysis and expressed as means ± SD (*p < 0.05, **p < 0.01).

The RNA sequence analysis of the virulent PmCQ2 (Figure 4A) and attenuated PmCQ6 (Figure 5A) *in vivo* and *in vitro* showed that a large number of DEGs were identified. The KEGG pathway analysis demonstrated that 13 KEGG pathways of the 104 matched showed significance (*P* < 0.05) in PmCQ2-m/PmCQ2 (Figures 4B,C and Supplementary Table 5), including ribosome, ABC transporters, microbial metabolism in diverse environments, tyrosine metabolism, carbon metabolism, ascorbate and aldarate metabolism, pentose and glucuronate interconversions, TCA cycle, and degradation of aromatic compounds and PTS, while the enriched KEGG pathways number was 11 (of 99 matched KEGG pathways) in PmCQ6-m/PmCQ6, including ribosome, ABC transporters, selenocompound metabolism, ascorbate and aldarate metabolism, biosynthesis of amino acids, arginine biosynthesis, alanine, aspartate and glutamate metabolism, methane metabolism, lysine degradation, and quorum sensing (Figures 5B,C and Supplementary Table 5). However, 46 virulence-related DEGs were significantly up-regulated in PmCQ2-m/PmCQ2, including eight capsular polysaccharide synthesis/transport, seventeen iron utilization, three LPS, two OMPs, one hemolysin, and fifteen others (Figure 4D and Supplementary Table 6), while in PmCQ6-m/PmCQ6, the number of virulence-related DGEs was only 38, including eight capsular polysaccharide synthesis/transport, ten iron utilization, two OMPs, one LPS, one hemolysin, and sixteen others (Figure 5D and Supplementary Table 6). To validate the results of the RNA sequence analysis, six capsular polysaccharide-related genes (*hexA/B/C/D, hyaB/C*), five iron utilization-related genes (*hemR, sitA, hitA, hutZ, fur*), three LPS-related genes (*lpxD, lpcA, lptA*), and one OMP-related gene (*ompA*) were used for qPCR analysis, which were similar to those obtained from the RNA-seq results in PmCQ2-m/PmCQ2 (Figures 4E,F) and in PmCQ6-m/PmCQ6 (Figures 5E,F).

### Virulence-Related DEGs of PmCQ2 and PmCQ6 *in vivo*

**Figure 6:**
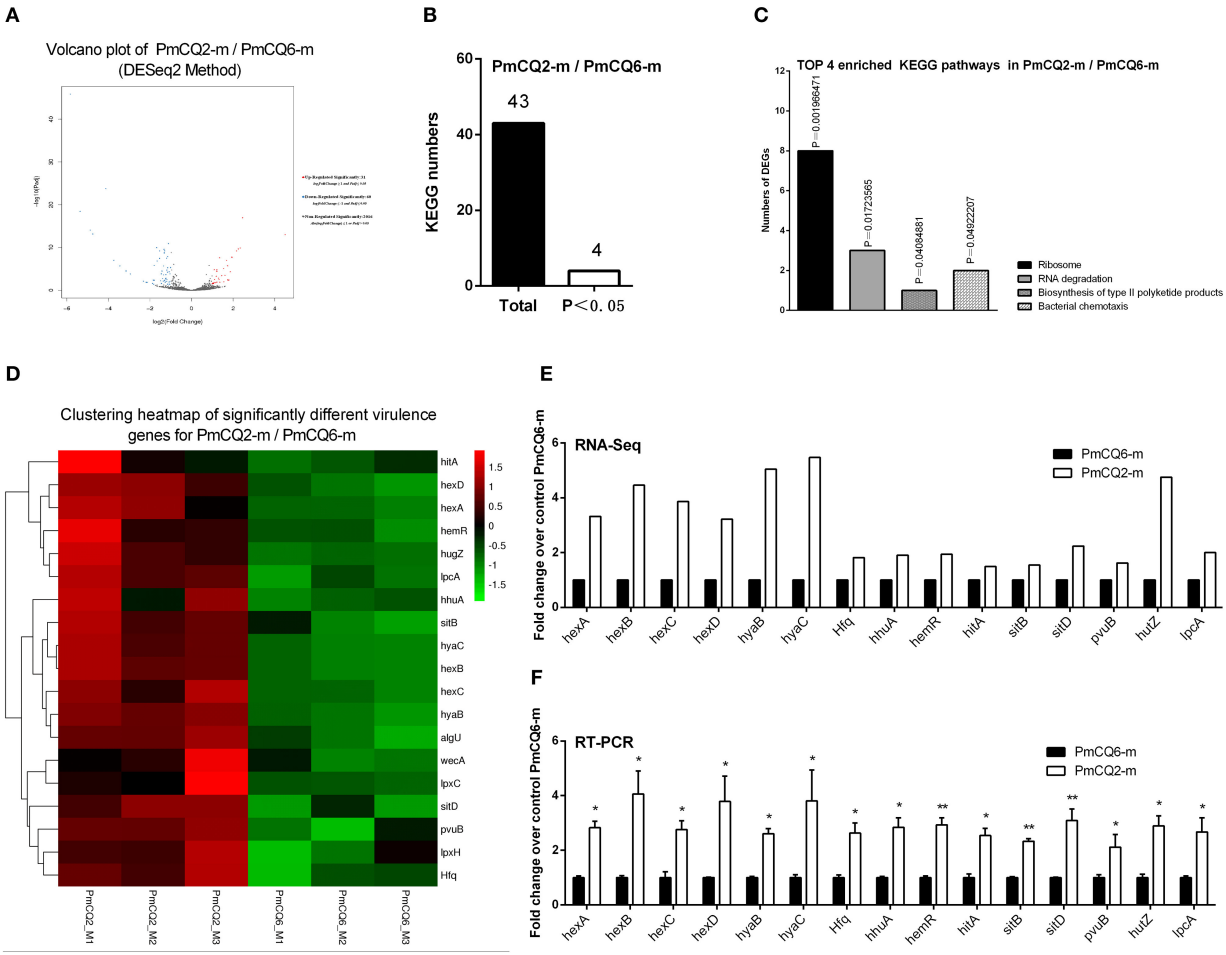
Virulence factor-related differentially expressed genes (DEGs) of PmCQ2-m compared to PmCQ6-m. **(A)** Volcano plot of PmCQ2-m/PmCQ6-m. **(B)** Total Kyoto Encyclopedia of Genes and Genomes (KEGG) numbers and significant KEGG numbers in PmCQ2-m/PmCQ6-m. **(C)** Top 4 enriched KEGG pathways of PmCQ2-m/PmCQ6-m, and the number of DEGs in each pathway is counted. **(D)** The clustering heat map of virulence factor-related DEGs in PmCQ2-m/PmCQ6-m. **(E)** The mRNA expression (RNA-Seq) of virulence factor-related DEGs in PmCQ2-m/PmCQ6-m (*n* = 3). **(F)** The mRNA expression (RT-PCR) of virulence factor-related DEGs in PmCQ2-m/PmCQ6-m (*n* = 3). These were representative of two independent experiments, with three replicates per group, analyzed by multiple comparative analysis and expressed as means ± SD (*p < 0.05, **p < 0.01).

To deepen the understanding on the virulence difference between the two strains, the expression profiles of PmCQ2 and PmCQ6 *in vivo* were analyzed; 91 DEGs were revealed (Figure 6A). By KEGG analysis, 43 KEGG pathways were matched, and 4 of them were significant (*P* < 0.05), which were mainly involved in ribosome, RNA degradation, biosynthesis of type II polyketide products, and bacterial chemotaxis *in vivo* (Figures 6B,C and Supplementary Table 5). 18 virulence-related DGEs were significantly up-regulated in PmCQ2-m/PmCQ6-m, including seven capsular polysaccharide synthesis/transport-related genes, seven iron utilization-related genes, one LPS-related gene, and three others (Figure 6D and Supplementary Table 6). To validate the results of the RNA sequence analysis, six capsular polysaccharide-related genes (*hexA/B/C/D, hyaB/C*), seven iron utilization-related genes (*hhuA, hemR, hitA, sitB/D, pvuB, hutZ*), and one LPS-related gene (*lpcA*) were chosen for qPCR analysis, which were similar to those obtained from the RNA-seq results (Figures 6E,F).

## Discussion

*Pasteurella multocida* serogroup A is an opportunistic pathogen causing severe respiratory diseases (32); among them, the virulent PmCQ2 and the attenuated PmCQ6 mainly cause pneumonia in cattle (16). It is well-known that the natural infectious route for *P. multocida* serotype A strain is through intranasal infection. Thus, in this study, a mouse model of *P. multocida* pneumonia caused by PmCQ2 (1 × 10^8^ CFU) and PmCQ6 (1 × 10^8^ CFU) was established *via* intranasal infection. The mice died within 72 h post-infection with PmCQ2, while only 40% died after PmCQ6 infection. The bacterial burden of PmCQ2 in mice lung tissue was significantly higher than that of PmCQ6, and PmCQ2 causes serious lung lesions (bronchitis, alveolar rupture, inflammatory cell infiltration, and some hemorrhage) than that of PmCQ6 in mice at 16 h post-infection. This result indicates that PmCQ2 is a virulent strain and PmCQ6 is an attenuated strain for mice as reported previously (16, 19). However, the underlying mechanism of the difference in virulence between PmCQ2 and PmCQ6 is still elusive. Thus, the whole gene expression profiles related to PmCQ2 and PmCQ6 *in vivo* and *in vitro* are explored with transcriptomic sequencing. In this study, we mainly studied the virulence factors of PmCQ2 and PmCQ6 *in vivo* and *in vitro*, such as capsule, iron, and OMPs.

Capsule is an important virulence factor of *P. multocida* serotype A associated with anti-phagocytic ability and serum resistance (10, 14, 33, 34), and the main component of capsule is hyaluronic acid (35). Ten capsule-associated genes have been identified in *P. multocida* type A, including *phyAB* (phospholipid substitution), *hyaBCDE* (capsule biosynthesis), and *hexABCD* (capsule transport) (4, 36). In this study, 32 up-regulated virulence-related DEGs in PmCQ2/PmCQ6, including five reported capsule-related DEGs (*hyaC* and *hexA/B/C/D*) and ten predicted capsule-associated DEGs *wza* (putative polysaccharide export protein YccZ precursor), *wzb* (cytoplasmic phosphatase), *wzc* (tyrosine protein kinase), *WbiI* (epimerase/dehydratase WbiI), *spsC* (spore coat polysaccharide biosynthesis protein spsC), *WeeH* (UDP-galactose phosphate transferase), *wecB* (UDP-N-acetylglucosamine 2-epimerase), *WbjC* (UDP-2-acetamido-2, 6-dideoxy-beta-L-talose-4-dehydrogenase), *fnlA* (UDP-glucose 4-epimerase), and *VipA* (Vi polysaccharide biosynthesis protein VipA/TviB). Moreover, the transcriptome data showed that there are 46 up-regulated virulence-related DEGs in PmCQ2-m/PmCQ2, including six reported capsule-related *hyaB/C* and *hexA/B/C/D* and two predicted capsule-associated genes *hscA* (capsule polysaccharide transporter) and *hscB* (capsule polysaccharide modification protein). Furthermore, 38 up-regulated virulence-related DEGs in PmCQ6-m/PmCQ6, including six reported capsule-related *hyaB/C* and *hexA/B/C/D* and two predicted capsule-associated genes *wza* (putative polysaccharide export protein YccZ precursor) and *oppF* (dipeptide transport system ATP-binding protein). In addition, there are 18 up-regulated virulence-related DEGs in PmCQ2-m/PmCQ6-m, including six reported capsule-related *hyaB/C* and *hexA/B/C/D* and capsular regulatory gene *Hfq* (37). The above-mentioned results indicate that the capsule-related virulence genes are important factors for the virulence difference between PmCQ2 and PmCQ6. Notably, the capsule of PmCQ6 was lower than that of PmCQ2 *in vitro*, and a comparative genomic analysis of the capsule-associated genes between PmCQ2 and PmCQ6 revealed that there was a single point mutation in the start codon of the *hyaC* gene. Thus, the point mutation of the start codon sequence of the *hyaC* may be the key reason for decreased capsule and virulence; more experiments are needed to explore.

Iron is necessary for bacteria to survive (38); genome-wide sequencing results indicated that more than 2.4% (53 genes) of the total genes in the PmCQ2 (23) (GenBank: CP033599.1) and over 2.1% (46 genes) (Supplementary Table 7) of the total genes in the PmCQ6 (GenBank accession number: CP033600.1) are predicted to encode proteins for iron uptake or acquisition. We found that the expression of six iron utilization-related DEGs *malK* (iron ABC transporter ATP-binding protein), *manB* (phosphomannomutase), *luxS* (S-ribosylhomocysteine lyase), *fbpC* (Fe^3+^ ions import ATP-binding protein FbpC), *pvuE* (iron-dicitrate transporter ATP-binding subunit), and *afuC* (Fe^3+^ ions transport system ATP-binding protein) were remarkably increased in PmCQ2/PmCQ6. Four TonB-receptor-related DEGs [*hmbR* (TonB-dependent hemoglobin receptor), *hemR* (TonB-dependent receptor), *hhuA* (TonB-dependent receptor), and *hemB* (TonB-dependent receptor)], seven iron utilization-related DEGs [*sitA* (iron-binding protein), *sitB* (iron ABC transporter permease), *fur* (transcriptional repressor of iron-responsive genes), *pvuB* (iron siderophore-binding protein), *hitA* (iron ABC transporter substrate-binding protein), *hitB* (iron ABC transporter permease), and *hitC* (Fe^3+^ irons ABC transporter, ATP-binding protein], and six heme utilization-related DEGs [*hutZ* (heme utilization protein HutZ), *hutX* (heme iron utilization protein), *hutC* (hemin ABC transporter permease), *hugZ* (heme iron utilization protein), *ccmF* (heme lyase subunit CcmF), and *hgpB* (hemoglobin-binding protein)] were significantly up-regulated in PmCQ2-m/PmCQ2. Two TonB-receptor-related DEGs (*hhuA* and *hemR*), six iron utilization-related DEGs (*pvuB, sitA, hitA, hitB, hitC*, and *fur*), and two heme utilization-related DEGs (*hutZ, hutX*) were obviously increased in PmCQ6-m/PmCQ6. Two TonB-receptor-related DEGs (*hhuA* and *hemR*), four iron utilization-related DEGs (*sitB/D, pvuB, hitA*), and one heme utilization-related DEG (*hutZ*) were especially up-regulated in PmCQ2-m/PmCQ6-m. These results suggest that iron uptake played an important contribution/role in the virulence difference of the two strains.

LPS is one of the most important factors associated with the pathogenesis of *P. multocida* (39, 40). Transcriptome sequencing results clarified that *lptA, lpxC* (N-acetylglucosamine deacetylase), *rfaF* (LPS heptosyltransferase II), and *rfaC/opsX* (LPS heptosyltransferase I) were significantly increased in PmCQ2/PmCQ6. Three LPS-related DEGs [*acpXL* (acyl carrier protein), *lpxD* (glucosamine N-acyltransferase), and *lpcA/gmhA* (phosphoheptose isomerase)] were significantly increased in PmCQ2-m/PmCQ2; *lptA* (lipid A ethanolamine phosphotransferase) was sharply increased in PmCQ6-m/PmCQ6, and *lpxC* was sharply upregulated in PmCQ2-m/PmCQ6-m. This result indicates that *lpxC* plays an important role in the virulence of PmCQ2.

As the main structure of the outer membrane of gram-negative bacteria, OMPs (e.g., membrane structural proteins, membrane transport proteins, membrane-bound proteins, and lipoproteins) play an important role in bacterial infection and pathogenic processes, such as adhesion, invasion, and intracellular survival (41–43). For example, OmpH and OmpA are the most typical virulence factors of *P. multocida* (44, 45). *OmpA* and *OmpP5* (outer membrane protein P5) were significantly upregulated in PmCQ2-m/PmCQ2 and PmCQ6-m/PmCQ6, and *OmpP5* was increased in PmCQ2-m/PmCQ6-m. This result indicates that *OmpP5* may be a key factor in the difference of virulence between the two strains.

In conclusion, the bacterial loads and lesions of lung tissue significantly decreased, and survival rates significantly improved in mice infected with PmCQ6 than that with PmCQ2. Notably, the differential transcription of virulence-related genes (e.g., capsule) is the main reason for the different pathogenicity of PmCQ2 and PmCQ6. This work provides new guidance for the mechanism study of the host–pathogen interactions of *P. multocida*.

## Data Availability Statement

The datasets generated in this study can be found in online repositories. The names of the repository/repositories and accession number(s) can be found below: https://www.ncbi.nlm.nih.gov/, PRJNA597831; https://www.ncbi.nlm.nih.gov/, PRJNA629108; https://www.ncbi.nlm.nih.gov/, PRJNA664837.

## Ethics Statement

The animal study was reviewed and approved by the Animal Ethics and Research Committee of Southwest University (Permit No.11-1025), Chongqing, China.

## Author Contributions

YP and PL designed the experiment. FH, ZZ, PL, XW, and LD conducted the experiment. FH, PL, RF, and NL analyzed the data and prepared the figures. FH and PL drafted the manuscript. YP, RF, NL, and PL revised and approved the final manuscript. All authors contributed to the article and approved the submitted version.

## Conflict of Interest

The authors declare that the research was conducted in the absence of any commercial or financial relationships that could be construed as a potential conflict of interest.

## Supplementary Material

Supplementary Table 1 qRT-PCR primers used in this work.

Supplementary Table 2 Transcriptome sequencing results statistics and quality assessment.

Supplementary Table 3 The total differentially expressed genes of all groups (fold change ≥2, *p*-value ≤ 0.05).

Supplementary Table 4 The levels of gene expression in all samples normalized with reads per kilobase of transcript per million reads mapped.

Supplementary Table 5 The complete Kyoto Encyclopedia of Genes and Genomes pathways annotated in all groups.

Supplementary Table 6 Virulent factors related differentially expressed genes of all groups (*P* ≤ 0.05).

Supplementary Table 7 The predicted 46 iron utilization-related genes of PmCQ6 based on whole genome sequencing.
